# Identification of *SMG3*, a QTL Coordinately Controls Grain Size, Grain Number per Panicle, and Grain Weight in Rice

**DOI:** 10.3389/fpls.2022.880919

**Published:** 2022-04-25

**Authors:** Ruosi Li, Zhen Li, Jing Ye, Yingying Yang, Juahua Ye, Siliang Xu, Junrong Liu, Xiaoping Yuan, Yiping Wang, Mengchen Zhang, Hanyong Yu, Qun Xu, Shan Wang, Yaolong Yang, Shu Wang, Xinghua Wei, Yue Feng

**Affiliations:** ^1^Chinese National Center for Rice Improvement and State Key Laboratory of Rice Biology, China National Rice Research Institute, Hangzhou, China; ^2^College of Agronomy, Shenyang Agricultural University, Shenyang, China; ^3^Institute of Crop and Nuclear Technology Utilization, Zhejiang Academy of Agricultural Sciences, Hangzhou, China

**Keywords:** grain size, grain number per panicle, grain weight, QTL, rice

## Abstract

Grain size, grain number per panicle, and grain weight are key agronomic traits that determine grain yield in rice. However, the molecular mechanisms coordinately controlling these traits remain largely unknown. In this study, we identified a major QTL, *SMG3*, that is responsible for grain size, grain number per panicle, and grain weight in rice, which encodes a MYB-like protein. The *SMG3* allele from M494 causes an increase in the number of grains per panicle but produces smaller grain size and thousand grain weight. The *SMG3* is constitutively expressed in various organs in rice, and the SMG3 protein is located in the nucleus. Microscopy analysis shows that *SMG3* mainly produces long grains by increasing in both cell length and cell number in the length direction, which thus enhances grain weight by promoting cell expansion and cell proliferation. Overexpression of *SMG3* in rice produces a phenotype with more grains but reduces grain length and weight. Our results reveal that *SMG3* plays an important role in the coordinated regulation of grain size, grain number per panicle, and grain weight, providing a new insight into synergistical modification on the grain appearance quality, grain number per panicle, and grain weight in rice.

## Introduction

Rice feeds nearly half of the world’s population. The yield per plant in rice is determined by four components: number of panicles, number of grains per panicle, grain weight, and ratio of filled grain (Li et al., 2013). The grain size of rice directly determines the grain weight, which in turn affects the yield of rice. In addition, grain size is an important appearance quality trait, which directly affects the milling quality of rice. Grain number per panicle is determined by the number of primary and secondary branches in rice (Xing and Zhang, 2010).

In recent years, using molecular markers to construct genetic linkage maps and QTL mapping for complex traits in rice has become a routine approach. Up to now, more than 500 QTLs related to rice grain size have been mapped,^1^ and several genes controlling grain size have been identified using natural variation populations. For example, *GS3* was the first cloned QTL responsible for grain size, which encodes a transmembrane protein containing four putative domains and negatively regulates grain length and grain weight (Mao et al., 2010). *GL3.1*/*qGL3* encodes a putative protein phosphatase (OsPPKL1) containing the Kelch repeat domains and regulates rice grain size and yield by influencing phosphorylation of Cyclin-T1;3 (Qi et al., 2012; Zhang et al., 2012; Gao et al., 2019). *GL7* was a major QTL for grain size in rice, and the tandem duplication of a 17.1-kb at the *GL7* locus leads to upregulation of *GL7* expression, thus increasing grain length and improved appearance quality of rice (Wang et al., 2015a,b). *GS9* encodes a protein without known conserved functional domain, which regulates grain shape by altering cell division (Zhao et al., 2018). *TGW2* was a novel grain size QTL, encoding the CELL NUMBER REGULATOR 1 (OsCNR1), that interacts with KRP1, a regulator of cell cycles in plants, to negatively regulate grain width and weight (Ruan et al., 2020). Recently, a novel miR167a-OsARF6-OsAUX3 module which regulates grain length and weight was reported in rice (Qiao et al., 2021).

In rice, several genes associated with grain number have been cloned and characterized (Ashikari et al., 2005; Kurakawa et al., 2007; Huang et al., 2009; Oikawa and Kyozuka, 2009; Tabuchi et al., 2011; Wu et al., 2016; Huo et al., 2017; Guo et al., 2018; Ren et al., 2018). Rice has a determinate inflorescence, in which inflorescence meristems initiate primary branch meristems that are attached to the central rachis of the inflorescence and then produce several secondary branch meristems (Zhang and Yuan, 2014). During panicle development, the primary and secondary branch meristems differentiate into spikelets, which determine the number of grains per panicle (Tabuchi et al., 2011; Guo et al., 2018). The spikelet hull consists of a palea and a lemma, which determine the final grain size in rice (Li and Li, 2016). However, the underlying molecular mechanisms coordinating grain size and grain number in rice remain largely unclear.

In this study, we detected fourteen QTLs affecting grain size, grain weight, and grain number per panicle on six chromosomes. In addition, we fine mapped and cloned of a QTL, *SMG3*, coordinately regulating grain size, grain number per panicle, and grain weight near the centromere on chromosome 3. We demonstrated that *SMG3* acted as a negative regulator of grain length, grain weight, and functioned as a positive regulator of grain number per panicle. These results laid a foundation for further genetic manipulation and trade-offs between grain size and grain number in rice.

## Materials and Methods

### Mapping Population and Field Experiments

The Indian small-grain rice M494 (*japonica*) and long-grain rice Zhong9 B (Z9B; *indica*) were both derived from the National Rice Mid-term Gene Bank of China. The F_2_ population consisting of 144 lines was developed from a cross between M494 and Z9B. Based on the QTL mapping results, we selected two long-grain lines in which *GS3* was fixed with homozygous M494, but the *SMG3* region was heterozygous in the F_2:3_ population. The two long-grain lines were backcrossed with M494 for three successive generations to obtain BC_3_F_2_ population. At first, 80 long-grain BC_3_F_2_ individual plants and 2,850 BC_3_F_3_ recessive single plants were genotyped with two markers Y3-53 and Y3-103 to fine map the grain size QTL-*SMG3* (Supplementary Table 1). Seven recombinant plants were further selected and selfed to produce BC_3_F_3_ lines for phenotyping and substitution mapping. Two sets of NIL-*SMG3* (NIL-*SMG3^M494^* and NIL-*SMG3^Z9B^*) were constructed (Supplementary Figure 1).

Field planting was carried out according to the conventional management mode of rice planting, the F_2_ population was planted in Fuyang experimental fields of China National Rice Research Institute (CNRRI; 30°32′ N, 120°12′ E) in the summer of 2016, and the F_2:3_ population was planted in Lingshui station of CNRRI (18°48′ N, 110°02′ E) in the winter of 2016. The BC_3_F_2_ population and NILs were planted in Fuyang experimental fields of CNRRI in the summer of 2019 and 2020. The BC_3_F_3_ lines were planted in Lingshui station of CNRRI in the winter of 2019.

The F_2_ and BC_3_F_2_ populations were planted with 20 cm between plants and 25 cm between rows. The BC_3_F_3_ lines and NILs were grown in a randomized complete block design with two replications, five rows per plot, and eight plants per row.

### Phenotyping

At mature stage, the seeds were individually harvested for phenotypic investigation. The full grains were selected, and thousand grain weight (TGW) was weighed on the electronic balance. Each single plant was weighed three times, and the average value was taken. Grain length (GL), grain width (GW), and grain number per panicle (GNP) were measured by an automatic seed counting and analyzing instrument (Model SC-G, Wanshen Ltd., Hangzhou, China). The grain length-width ratio (LWR) is equal to GL divided by its GW. In addition, the percentage of chalky grains and degree of chalkiness of milled rice were measured using a grain appearance analyzer instrument (Model SC-E, Wanshen Ltd., Hangzhou, China).

### Microscopy Observations

For glume cell observation, the spikelets of NIL-*SMG3^M494^* and NIL-*SMG3^Z9B^* were collected at maturity stage. The samples were fixed in FAA solution (formalin: glacial acetic acid: ethanol in 1:1:18 ratio by volume) at 4°C for 24 h, then were dehydrated and dried as described by Feng et al. (2021). The outer and inner surfaces of the spikelet glumes were observed under the scanning electron microscope (Hitachi, S-3400).

### RNA Extraction and qRT-PCR

The RNA was extracted from various plant tissues using a MiniBEST Plant RNA Extraction Kit (Takara Bio Inc., Japan). First-strand cDNAs were synthesized using Prime Script RT Master Mix (TAKARA Bio Inc.). Quantitative RT-PCR analysis was performed on the Applied Biosystems 7,500 Real-Time PCR system with a 2 × SYBR Green PCR Master Mix (Applied Biosystems). The rice *Actin* gene was used as an internal control. Each measurement was replicated at least three times with three biological samples. The qRT-PCR primers used in these assays are presented in Supplementary Table 1.

### Vector Construction and Plant Transformation

For complementation test of *SMG3*, using the high-purity DNA extracted from the NIL-*SMG3^M494^* as the template, the gene-specific primers were used to amplify the DNA fragment, including the 2-kb region upstream of the transcription start site and 1.5-kb downstream of the termination site of *SMG3*/*LOC_Os03g29614*, and cloned into the pCAMBIA1300 vector to generate *pSMG3::SMG3^M494^* plasmid. The plasmid was introduced into the callus of NIL-*SMG3^Z9B^* by *Agrobacterium*-mediated transformation.

To generate the overexpression construct, the full-length coding sequence (CDS) of *LOC_Os03g29614* was amplified from NIL-*SMG3^M494^* and cloned into the vector pCAMBIA1300S. The plasmid was introduced into the callus of Nipponbare by *Agrobacterium*-mediated transformation.

To analyze the expression pattern of *SMG3*, a 2072-bp promoter fragment was cloned from NIL-*SMG3^M494^* and was introduced into the pCAMBIA1301 vector. The constructed plasmid *SMG3::GUS* was then transformed into the rice variety Nipponbare by the *Agrobacterium*-mediated method. GUS staining was performed on some parts (root, base, stem, node, flag leave, pulvinus, leaf sheath, young panicle, and flowering panicle) of positive plants (Jefferson, 1989). After decolorization, several parts were placed in the scanner for scanning and observation. The primers used are listed in Supplementary Table 1.

### Subcellular Localization of SMG3

To determine the subcellular localization of SMG3, the *Pro35S::SMG3-GFP* plasmid was introduced into the Agrobacterium tumefaciens GV3101 and injected into rice protoplasts. The nuclear protein GHD7 fused with RFP was used as a nuclear marker (Xue et al., 2008; Fu et al., 2019). GFP signal was observed by confocal laser microscope. The primers are listed in Supplementary Table 1.

### Data Analysis

Genetic map was constructed using Mapmaker/EXP version 3.0 of SSR marker genotype data. The Kosambi Mapping Function was used to convert the recombination frequency to cM. The QTLs were named as described by McCouch et al. (1997).

All data were collected from at least three independent biological replicates and presented as means ± SD. Statistical analysis was performed using the SAS version 9.2. Statistical significance was determined using Student’s *t*-test for comparison of the two groups.

## Results

### Fine Mapping of *SMG3*, a QTL for Grain Size, and Grain Weight

**Figure 1:**
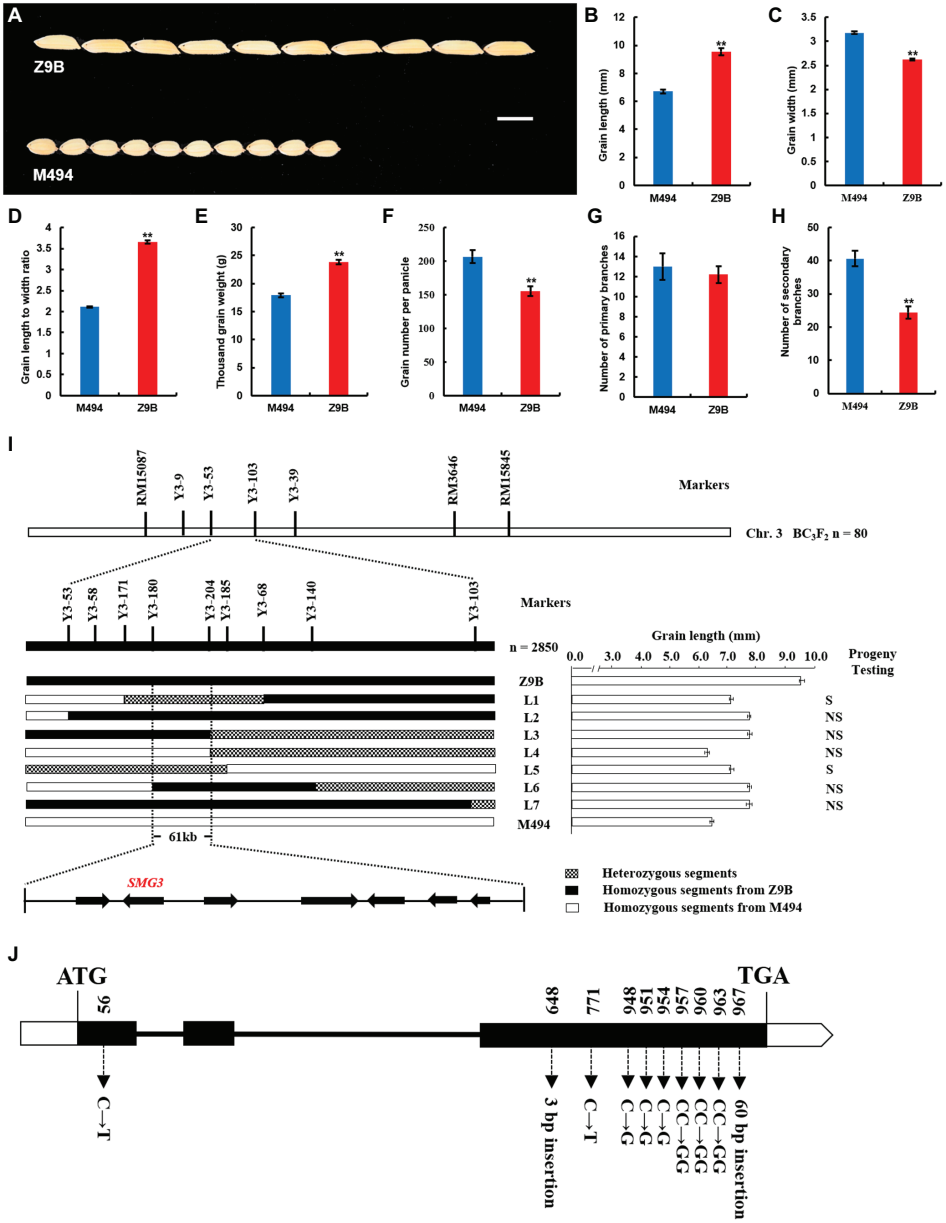
Map-based cloning of *SMG3*. **(A)** Z9B and M494 rice grains. Scale bar: 6 mm. **(B–H)** Comparison of Z9B and M494 grain length (*n* = 20; **B**), grain width (*n* = 20; **C**), grain length to width ratio (*n* = 20; **D**), thousand grain weight (*n* = 20; **E**), grain number per panicle (*n* = 20; **F**), number of primary branches (*n* = 20; **G**), and number of secondary branches (*n* = 20; **H**). Data are given as means ± SD. ^**^indicates *p* < 0.01 by Student’s *t*-test. **(I)** Fine mapping of *SMG3* and candidate gene analysis. S, segregation; NS, no segregation. **(J)** Gene structure and allelic variations of *LOC_Os03g29614* (*SMG3*) between Z9B and M494. Filled boxes represent exons, black lines represent introns. The start codon (ATG) and the stop codon (TGA) are indicated.

The M494 and Z9B differed significantly in grain size, grain weight, and grain number (Figures 1A–H). To dissect the genetic basis of these traits, we conducted a quantitative trait locus (QTL) analysis using an F_2_ population derived from a cross between M494 and Z9B. Fourteen QTLs affecting grain size, grain weight, and grain number per panicle (GNP), including one for grain length (GL), six for grain width (GW), three for grain length to width ratio (LWR), one for thousand grain weight (TGW), and three for GNP, were identified in this population (Supplementary Table 2).

On the chromosome 3, we found there were two LOD peaks for grain size and weight at approximately 79 cM and 92 cM in the F_2_ population (Supplementary Figure 2), which suggested that there existed two QTLs for grain size and weight in the 79–92 cM region of chromosome 3. One peak indicated that is the previously cloned grain length gene *GS3*, and the other might be a novel QTL for grain size and weight closely linked to *GS3*. Therefore, first, we use the functional marker SF28 of *GS3* gene to screen out the individual plant with genotype A of *GS3* and a long-grain phenotype relative to M494 in the F_2:3_ population (Fan et al., 2009). Then, we constructed a BC_3_F_2_ population with M494 as a recurrent parent in the target region flanked by RM15087 and RM3646. The QTL named *SMG3* for grain length was mapped to the region between the STS marker Y3-53 and the Indel marker Y3-103 using 80 long-grain BC_3_F_2_ individual plants and their progenies. To fine map the position of the *SMG3* locus, we selected 2,850 BC_3_F_3_ individuals heterozygous between marker Y3-53 and Y3-103. Seven recombinants were identified, which were genotyped with seven newly designed indel markers, progenies exhibiting long-grain phenotype (Supplementary Table 1). A precise phenotyping of grain length was performed by progeny testing, which also enabled the determination of segregation patterns of *SMG3* in each recombinant line. The L4 progeny line showed short grain length, indicating that the L4 recombinant line was M494 homozygous for *SMG3*. The L2, L3, L6, and L7 progeny lines at *SMG3* locus were Z9B homozygous due to their progenies exhibited long-grain phenotype. The L1 and L5 progeny lines displayed segregating grain length, indicating that the L1 and L5 recombinant lines were heterozygous at *SMG3* locus (Figure 1I). These results allowed us to delimite *SMG3* to a region of approximately 61 kb between the markers Y3-180 and Y3-204.

In the 61 kb target region, seven genes are annotated. Among these genes, *LOC_Os03g29600* encodes a putative transposon protein, *LOC_Os03g29614* encodes a protein containing a putative myb-like DNA-binding domain, *LOC_Os03g29630* encodes an ulp1 protease family protein, *LOC_Os03g29650* and *LOC_Os03g29660* encode putative retrotransposon proteins, *LOC_Os03g29680* encodes a putative early flowering protein, and *LOC_Os03g29690* encodes a putative expressed protein. Interestingly, the *LOC_Os03g29614* encodes the myb-related protein OsMYB3 and was identified as a negative regulator of grain length (Suzuki et al., 1997; Li et al., 2020), which suggested that the *LOC_Os03g29614* is the most likely candidate gene for *SMG3*.

### Confirmation of *SMG3* Function

The *LOC_Os03g29614* gene contains 3 exons and 2 introns (Figure 1J). We sequenced the *LOC_Os03g_29614* gene in the M494 and Z9B varieties. Comparative analysis of the coding regions of the *LOC_Os03g_29614* gene revealed that the M494 contains eight polymorphisms compared with Z9B, including one nucleotide substitution (c.56 C to T) in the exon 1, one 3 bp insertion mutation (c.648 - to GCA), one synonymous mutation (c.771 C to T), six missense polymorphisms (c.948 C to G, c.951 C to G, c.954 C to G, c.957 CC to GG, c.960 CC to GG, and c.963 CC to GG) and a 60 bp insertion in the exon 3 (Figure 1J). The novel 60 bp insertion in the exon 3 of M494 resulted in a frame-shift mutation and may influence the grain size, grain weight and grain number per panicle.

**Figure 2:**
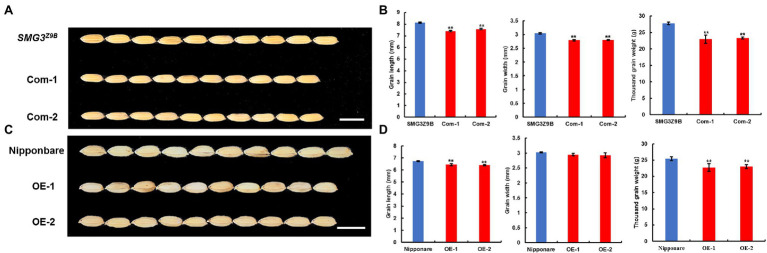
Complementary test and overexpression analysis. **(A)** Grains of NIL-*SMG3^Z9B^* and complementary (Com) transgenic plants. Scale bar: 8 mm. **(B)** Grain length, grain width, and thousand grain weight of NIL-*SMG3^Z9B^* and the complementary transgenic lines. **(C)** Grains of Nipponbare and overexpression (OE) transgenic plants. Scale bar: 8 mm. **(D)** Grain length, grain width, and thousand grain weight of Nipponbare and the *SMG3* overexpression transgenic lines. Data in **(B)** and **(D)** are the means ± SD; ^**^ indicates *p* < 0.01 by Student’s *t*-test.

To confirm whether the *LOC_Os03g_29614* gene was the candidate gene for *SMG3*, we carried out a genetic complementation test. A NIL-*SMG3^M494^* DNA fragment containing the 2-kb promoter region, the entire *LOC_Os03g_29614* gene, and 1.5-kb downstream of the gene was cloned into the binary vector pCAMBIA1300 to generate the *pSMG3* construct. The *pSMG3* construct was transferred into the NIL-*SMG3^Z9B^* background. The positive transgenic plants produced shorter grains and reduced TGW compared with the NIL-*SMG3^Z9B^* (Figures 2A,B). In addition, we overexpressed NIL-*SMG3^M494^* in the Nipponbare background and found that the *SMG3*-overexpressing plants exhibited a decrease in GL and TGW but increase in GNP (Figures 2C,D; Supplementary Figure 3). Our results indicated that the *SMG3* gene is a new allele of *OsMYB3*, which functioned as a negative regulator for grain size and grain weight, but also as a positive regulator of GNP. This finding showed that the Indian variety M494 possessed a desirable allele of *OsMYB3*, which provided a valuable resource for rice genetics research and breeding.

### *SMG3* Increases Grain Size and Grain Weight but Decreases Grain Number

**Figure 3:**
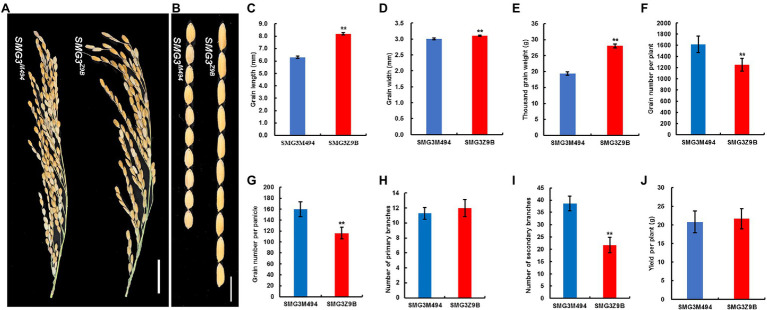
Comparison of the agronomic traits between NIL-*SMG3^Z9B^* and NIL-*SMG3^M494^*. **(A)** Panicle phenotype of NIL-*SMG3^Z9B^* and NIL-*SMG3^M494^*. Scale bar: 3 cm. **(B)** NIL-*SMG3^Z9B^* and NIL-*SMG3^M494^* rice grains. Scale bar: 8 mm. **(C–J)** Grain length **(C)**, grain width **(D)**, thousand grain weight **(E)**, grain number per plant **(F)**, grain number per panicle **(G)**, number of primary branches **(H)**, number of secondary branches **(I)**, and yield per plant **(J)** of NIL-*SMG3^Z9B^* and NIL-*SMG3^M494^*. Data are given as means ± SD. ^**^ Indicates *p* < 0.01 by Student’s *t*-test.

To clarify the function of *SMG3*, we developed two near-isogenic lines (NILs) differing only at the *SMG3* region. The NILs carrying the M494 and Z9B alleles at the *SMG3* locus were designated NIL-*SMG3^M494^* and NIL-*SMG3^Z9B^*, respectively. Compared with NIL-*SMG3^Z9B^*, NIL-*SMG3^M494^* showed significantly decreased GL, GW, and TGW, increased grain number per plant, GNP, and the number of secondary branches (SB; Figures 3A–G,I). However, no significant differences were observed between the NILs in number of primary branches and grain yield per plant (GYP; Figures 3H,J). These results suggest that *SMG3* plays an important role in coordinated regulation between grain size and grain number.

### *SMG3* Regulates Grain Size by Promoting Cell Expansion and Cell Proliferation

**Figure 4:**
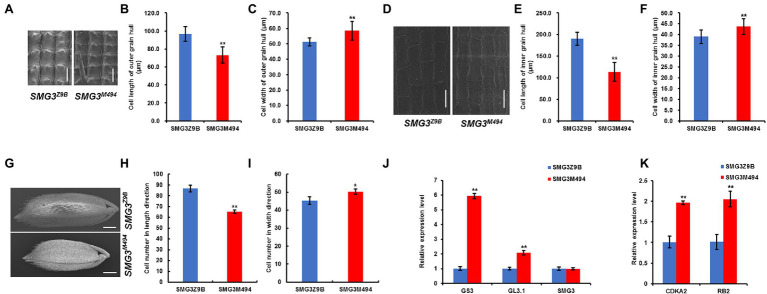
Histological and qPCR analysis between NIL-*SMG3^Z9B^* and NIL-*SMG3^M494^*. **(A)** Scanning electron microscope analysis of the outer surfaces of the grain hulls in *SMG3^Z9B^* and *SMG3^M494^*. Scale bar: 50 μm. **(B,C)** The average length and width of outer epidermal cells. **(D)** Scanning electron microscope analysis of the inner surfaces of the grain hulls in NIL-*SMG3^Z9B^* and NIL-*SMG3^M494^*. Scale bar: 50 μm. **(E,F)** The average length and width of inner epidermal cells. **(G)** Scanning electron micrographs of the outer surfaces of glumes in NIL-*SMG3^Z9B^* and NIL-*SMG3^M494^*. Scale bar: 1 mm. **(H,I)** the cell number in the outer glumes along the length and width direction between NIL-*SMG3^Z9B^* and NIL-*SMG3^M494^*. **(J)** qPCR analysis of *GS3*, *GL3.1*, and *SMG3* genes in the NIL-*SMG3^Z9B^* and NIL-*SMG3^M494^*. **(K)** qPCR analysis of *CDKA2* and *RB2* genes between NIL-*SMG3^Z9B^* and NIL-*SMG3^M494^*. Data are given as means ± SD. ^*^ and ^**^ indicate *p* < 0.05 and *p* < 0.01 by Student’s *t*-test.

In rice, grain size is restricted by the size of the spikelet hull (Song et al., 2015), which is controlled by the coordination of cell proliferation and expansion (Duan et al., 2014). Therefore, we examined the cell size and cell number in the spikelet hulls of the NIL-*SMG3^M494^* and NIL-*SMG3^Z9B^* using the scanning electron microscope. Compared with NIL-*SMG3^Z9B^*, the average cell length of the outer and inner glumes was decreased in NIL-*SMG3^M494^*, but the average cell width was increased (Figures 4A–F). In addition, we measured the cell number of outer spikelet hulls along the length and width between NIL-*SMG3^Z9B^* and NIL-*SMG3^M494^*. Compared with NIL-*SMG3^Z9B^*, the total cell number along the length was significantly reduced in the NIL-*SMG3^M494^*, but the cell number along the width was significantly increased in the NIL-*SMG3^M494^* (Figures 4G–I). These results suggested that *SMG3* affects grain size by promoting cell expansion and cell proliferation in spikelet hulls.

Several genes/QTLs, such as *GL7*/*GW7* and *GS2*, are known to control grain size by regulating cell expansion, and *GS3*, *GL3.1*/*qGL3*, and *GS5* are involved in regulation of cell proliferation. To reveal how *SMG3* regulates cell expansion and cell proliferation in spikelet hulls, we detected their expression levels in the NIL-*SMG3^M494^* and NIL-*SMG3^Z9B^* panicles. Compared with NIL-*SMG3^Z9B^*, only the expression levels of *GS3* and *GL3.1*/*qGL3* were significantly raised in the NIL-*SMG3^M494^* (Figure 4J), and the expression levels of *GL7*/*GW7*, *GS2*, and *GS5* were similar between NIL-*SMG3^M494^* and NIL-*SMG3^Z9B^* (data not shown). A similar difference was observed between Nipponbare and the *SMG3* overexpression plants (Supplementary Figure 4).

Furthermore, we also detected the expression of genes involved in the cell cycle, such as *CYCA2.1*, *CDKA2*, *CYCD4.2*, *CDKC1*, *CYCU3.1*, and *RB2*, and found that besides *CYCA2.1*, *CYCD4.2*, *CDKC1*, and *CYCU3.1* (data not shown), *CDKA2* and *RB2* were downregulated in NIL-*SMG3^Z9B^*, suggesting that the increase in cell length in NIL-*SMG3^Z9B^* might result from lower expression of these two genes promoting cell proliferation (Figure 4K). In addition, we found that the *CDKA2* and *RB2* expression levels were upregulated in the *SMG3* overexpression plants compared with Nipponbare (Supplementary Figure 5). Taken together, these results supported that *SMG3* regulated grain size by promoting cell expansion and cell proliferation in spikelet hulls.

### The *SMG3* Allele of M494 Could Improve Grain Appearance Quality of Milled Rice

**Figure 5:**
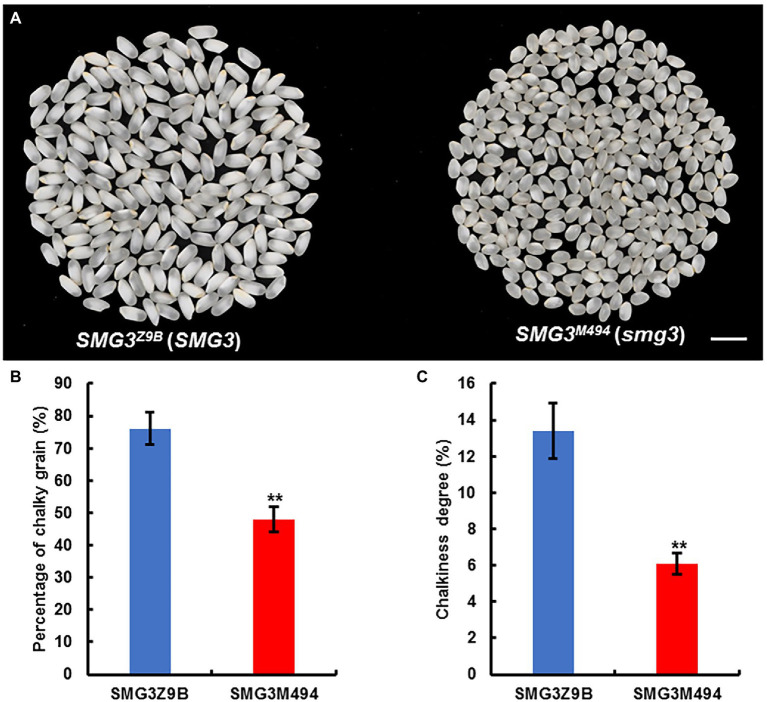
Improved appearance quality of milled rice by introgression of *SMG3* allele from M494. **(A)** Comparison of the appearance of milled rice between NIL-*SMG3^Z9B^* and NIL-*SMG3^M494^*, carrying the homozygous for Z9B and M494 allele, respectively. Scale bar: 8 mm. **(B,C)** The percentage of milled rice with chalkiness **(B)** and the chalkiness degree of milled rice **(C)** from NIL-*SMG3^Z9B^* and NIL-*SMG3^M494^*. Data are given as means ± SD. ^**^ Indicates *p* < 0.01 by Student’s *t*-test.

Although the grain size and grain weight of NIL-*SMG3^M494^* were smaller than NIL-*SMG3^Z9B^*, the final grain yield per plant was identical (Figure 3J). As expected, milled rice from NIL-*SMG3^Z9B^* was more slender than that of NIL-*SMG3^M494^* (Figure 5A). More importantly, the chalky grain percentage and chalkiness degree of milled rice of NIL-*SMG3^M494^* were significantly decreased than NIL-*SMG3^Z9B^* (Figures 5B,C). These findings suggested that the potential for application of the *SMG3* allele of M494 to improve grain appearance quality in high yield but low quality rice varieties.

### The Expression Pattern and Subcellular Localization of *SMG3*

**Figure 6:**
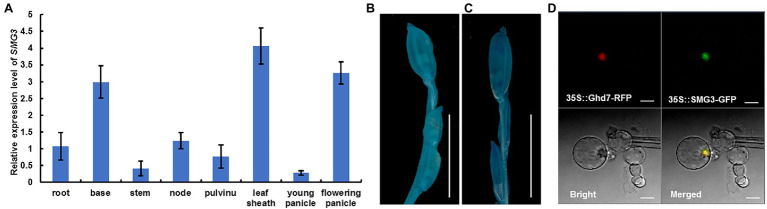
Expression pattern of *SMG3* and subcellular localization of SMG3 protein. **(A)** Relative expression levels of *SMG3* in different tissues analyzed by qRT-PCR. Values are means ± SD of three independent experiments. **(B,C)** Histochemical analysis of GUS activity in caryopsis of 4–5 cm **(B)** and 13–14 cm **(C)** long panicle. Scale bars: 1 cm. **(D)** Subcellular localization of the SMG3 protein by GFP assays. The subcellular localization was performed with red fluorescent protein (RFP) as a nuclear marker. The 35S::SMG3-GFP and 35S::Ghd7-RFP were co-expressed in rice protoplasts. Scale bar: 10 μm.

To examine the temporal and spatial expression pattern of *SMG3*, total RNA from nine organs of the *SMG3::GUS* transgenic plants, including root, base, stem, node, flag leave, pulvinus, leaf sheath, young panicle, and flowering panicle was extracted. qRT-PCR analysis showed that *SMG3* was constitutively expressed in various rice organs, with high expression in the leaf sheath, base, and flowering panicle (Figure 6A). In the *SMG3::GUS* transgenic plants, GUS activity of old panicle was slightly stronger than in young panicle (Figures 6B,C). The expression pattern of *SMG3* is consistent with its role in spikelet hull development.

To determine the subcellular localization of SMG3, we constructed SMG3::GFP and Ghd7-RFP fusions driven by the 35S promoter. The two fusions were transferred together into rice protoplasts. We observed that the SMG3::GFP fusion protein was localized in the nucleus of rice protoplasts (Figure 6D). This result indicated that *SMG3* encodes a nuclear protein, consistent with its proposed role as a transcription factor (Li et al., 2020).

## Discussion

### *SMG3* Acts as a Negative Regulator of Grain Length and Grain Weight, but a Positive Regulator of Grain Number per Panicle

Grain size and weight are important determinants of yield production and appearance quality in rice. Several genes regulating grain size and weight such as *GS3*, *GS5*, *GSE5*, *GW2*, *GS2*, *GL7/GW7*, and *TGW2* (Song et al., 2007; Mao et al., 2010; Li et al., 2011; Hu et al., 2015; Wang et al., 2015a,b; Duan et al., 2017; Ruan et al., 2020) have been identified, but the molecular mechanisms underlying grain size and weight control in rice remain elusive. In the present study, we reported QTL mapping results for grain size, grain weight, and grain number per panicle with an F_2_ population from a cross between a *japonica* small-grain variety (M494) and a long-grain *indica* variety (Z9B). A total of fourteen putative QTLs for grain size, grain weight, and grain number per panicle were detected in the F_2_ population (Supplementary Table 2). Especially, the main QTL cluster on chromosome 3 was responsible for GL, GW, LWR, and TGW in the F_2_ population, which covers two QTL peaks, one is the previously cloned grain length gene *GS3*, and the other is the QTL for grain length and weight, *SMG3* (Supplementary Figure 2). To fine map the hidden QTL-*SMG3* nearby *GS3*, we generated a large BC_3_F_3_ population and selected recombinant individuals in the region around *SMG3*. Using a chromosome fragment substitution strategy, the *SMG3* gene was narrowed down to a 61 kb region. Genetic complementation test demonstrated that *SMG3* is a novel allele of *OsMYB3*. Interestingly, The NIL-*SMG3^M494^* showed a significant increase in GNP compared to NIL-*SMG3^Z9B^*, and similar differences were observed between *SMG3* overexpression positive and negative plants (Figure 3G; Supplementary Figure 3). Our results demonstrated *SMG3* was a negative regulator of GL and grain weight but a positive regulator of GNP. This finding was consistent with the previous study that showed that an increase in GNP usually causes a decrease in grain size and weight (Huang et al., 2009).

### *SMG3* Promotes Cell Expansion and Cell Proliferation

Grain size is determined by cell proliferation and cell expansion (Duan et al., 2014). Our results revealed that the length of epidermal cells of the outer and inner glumes was increased in NIL-*SMG3^Z9B^* compared with those in NIL-*SMG3^M494^*, and the total cell number along the length direction was also significantly increased in the NIL-*SMG3^Z9B^* (Figures 4B,E,H). In addition, we found that two cell cycle genes, namely, *CDKA2* and *RB2*, were upregulated in NIL-*SMG3^M494^* compared with NIL-*SMG3^Z9B^* (Figure 4K). The previous studies showed that RB2 could bind to E2 promoter binding factor (E2F) and inhibits the activity of E2F transcription factor, preventing cell cycle progression (Dyson, 1998; Desvoyes et al., 2006; Shi et al., 2019), and the high level expression of *RB2* which was in the NIL-*SMG3^M494^* may inhibit cell elongation and proliferation in the length direction in spikelet hulls and result in small grains in the present study. As is known, Cylin-Dependent Kinase (CDK)-related genes play functional roles in cell cycle regulation of seed size and development in plants (Jiang et al., 2012; Sabelli et al., 2013; Dante et al., 2014; Chen et al., 2015; Li et al., 2019; Liu et al., 2021). In this study, downregulation of *CDKA2* may contribute to increase in cell expansion and proliferation in the length direction in spikelet hulls and lead to longer grains in NIL-*SMG3^Z9B^*. Collectively, our results suggested that *SMG3* regulated grain size by promoting both cell expansion and cell proliferation.

### Application of the Novel Allele of *SMG3* in Rice Breeding

Cereal crops had been selected and domesticated to satisfy human needs (Sang, 2009). However, the process usually led to a decrease in offspring genetic diversity, and lost some useful genetic loci during domestication. We analyzed the 120 rice re-sequenced varieties by Li et al. (2020) and found that the 60 bp insertion at nucleotide 967 in exon 3 was specific in the M494 accession, which indicated that it was a novel mutation of *OsMYB3* and might not have been selected by breeders. Compared with *SG3* (Li et al., 2020), *SMG3* is a new allele of *OsMYB3*, and not only negatively affected grain length and grain weight, but positively regulated grain number per panicle. In addition, our results showed that the NIL-*SMG3^M494^* carrying the M494 allele could reduce the chalky grain percentage and chalkiness degree of milled rice compared to NIL-*SMG3^Z9B^*, and the final grain yield per plant was equivalent. These results revealed that the M494 allele of *SMG3* has the potential for grain quality improvement and could be used to combine with other genes for grain size to regulate rice grain appearance in breeding.

## Data Availability Statement

The datasets presented in this study can be found in online repositories. The names of the repository/repositories and accession number(s) can be found in the article/Supplementary Material.

## Author Contributions

RL, ZL, SW, XW, and YF conceived and designed the experiments. RL, ZL, JY, YiY, JL, YW, and MZ performed the experiments. HY, QX, SW, and YaY analyzed the data. RL and YF wrote the manuscript and other authors revised the manuscript. All authors contributed to the article and approved the submitted version.

## Funding

This study was supported by the Key Technology Research and Development Program of Zhejiang Province (2021C02056), the Key Research and Development Project of Hainan Province (ZDYF2021XDNY170), the Fundamental Research Funds for Central Public Welfare Research Institutes of Chinese Rice Research Institute (CPSIBRF-CNRRI-202101), and the Science and Technology Innovation Program of Chinese Academy of Agricultural Sciences (CAAS-ASTIP-2013-CNRRI).

## Conflict of Interest

The authors declare that the research was conducted in the absence of any commercial or financial relationships that could be construed as a potential conflict of interest.

## Publisher’s Note

All claims expressed in this article are solely those of the authors and do not necessarily represent those of their affiliated organizations, or those of the publisher, the editors and the reviewers. Any product that may be evaluated in this article, or claim that may be made by its manufacturer, is not guaranteed or endorsed by the publisher.

## Supplementary Material

The Supplementary Material for this article can be found online at https://www.frontiersin.org/articles/10.3389/fpls.2022.880919/full#supplementary-material

## Footnotes

^1^ http://www.gramene.org/

## Abbreviations

QTL: Quantitative Trait Locus
Z9B: Zhong 9B
MYB: Myeloblastosis
NIL: Near-Isogenic Line
GL: Grain Length
GW: Grain Width
LWR: Grain Length-Width Ratio
TGW: Thousand Grain Weight
GNP: Grain Number per Panicle
cM: Centimorgan
RT-PCR: Reverse Transcription Polymerase Chain Reaction
DNA: Deoxyribo Nucleic Acid
RNA: Ribo Nucleic Acid
GFP: Green Fluorescent Protein
GUS: *β*-Glucuronidase
CDS: Coding Sequence
SB: Secondary Branches
CDK: Cylin-Dependent Kinase
