# Supplementary material for: Identification of SMG3, a QTL Coordinately Controls Grain Size, Grain Number per Panicle, and Grain Weight in Rice
Source: Front Plant Sci. 2022 Apr 25;13:880919. doi: 10.3389/fpls.2022.880919 (PMC9085218; doi:10.3389/fpls.2022.880919)
Supplement: Supplementary file 1 [file Table_1.DOCX]

Supplementary Table 1 Primers used in this study

| Primers | Forward 5’- 3’ | Reverse 5’- 3’ | Purpose |
| --- | --- | --- | --- |
| SF28 | TGCCCATCTCCCTCGTTTAC | GAAACAGCAGGCTGGCTTAC | mapping |
| RM15087 | CACCTCGTTCCTACGTCATATTGC | GGCGGCATAGTAGCGTTTATAGG | mapping |
| RM3646 | ACTAGAGCACCCTCGCTGAG | CTCAGCCACCCCATCAAC | mapping |
| RM15845 | CCCTCCTCCGCCTACAAATACGC | ATGCGGAGGTCGAAGCCCTAGC | mapping |
| Y3-9 | CACCGTTTGGTTTGGCAG | AGCATGGTGTACCTCGTCG | mapping |
| Y3-39 | GGAGGATGTAATTTGGATGA | CATCCTCGATACGATGTTTT | mapping |
| Y3-53 | ATCACCCTTTTCCCAACTAT | GTCCAACTGTAGGCAAAAAC | mapping |
| Y3-103 | AAGAACCCACCTGCGGTTAGC | CTACAGCTTTCTTGATTCGCTTGG | mapping |
| Y3-58 | AAACTGGTCAAAATTAAGTAAA | TGATAATTACTACTCCCTCCA | mapping |
| Y3-68 | GGGCATTATAGTCATTTCCA | AAAAGGAAAGTTTGATATGGAG | mapping |
| Y3-140 | TATGTGGGCTCCACTTTACT | CTATGAATACCCAACCTCCA | mapping |
| Y3-171 | TTCCGTCTCATAAACAACCT | GTACCGTACTCTCTCCGTTC | mapping |
| Y3-180 | ATATGCAGATCAATCAACGG | TAATTTGCAATGGTGTTCAA | mapping |
| Y3-185 | TACTCCGATGAAATCCATGT | AAACTGGGATGGAGAAAGTAG | mapping |
| Y3-204 | AATTCCTGCAATTTATTCCC | CTTGGTTCTTTTAAAGTGCG | mapping |
| *Comp-1* | ATTCGAGCTCGGTACCTAAAAGAAATAGTTGCCCATCA | GAATAGAAAGCTTCATTCCATCATGTGCTAAAGAGAG | vector construction |
| *Comp-2* | TAGCACATGATGGAATGAAGCTTTCTATTCGTTTCTC | GCCTGCAGGTCGACTCTAGAAGAGCAGCATATTAGCAC | vector construction |
| *OE* | AGCTTTCGCGAGCTCGGTACCATGGGGAGGAAGCCGTGC | TGCCTGCAGGTCGACTCTAGATCAGAGGAGCCATGGCGC | vector construction |
| *GUS* | TTCGAGCTCGGTACCTAAAAGAAATAGTTGCCCATC | CTCAGATCTACCATGCCACGATTGCTCGATCGAT | vector construction |
| GFP | TCGATACCGTCGACCTCGAGATGGGGAGGAAGCCGTGC | TGCTCACCATGGTACCGAGGAGCCATGGCGC | vector construction |
| *Actin* | ATCCATCTTGGCATCTCTCAGC | CACAATGGATGGGCCAGACT | qRT-PCR |
| *SMG3* | TGGTGTCCTACATCGCCAAG | ATGTTGCCCCTCTTGATCCC | qRT-PCR |
| *GS3* | TGACGAATTCATCGGAAGAACT | ACTTCTTCAAGAAGTGGTGAGA | qRT-PCR |
| *GL3.1* | GGAGGTGGTCGGATGGTAGA | TCTTCCTATCCTGGGAGTTG | qRT-PCR |
| *GS5* | GTTCTCGGTACTGCGTGGAAG | ACTCCACAAACCTCCCAGCA | qRT-PCR |
| *GS2* | TGCGTCCCTTCTTTGATGAGT | ACAGTTGGGTGCCTGAGAATG | qRT-PCR |
| *GL7* | GGGACACCGGAGGCCTTA | TGCCCATTCCTCCTTGCAT | qRT-PCR |
| *CYCA2.1* | GGACACTTGACCAATCTGAC | TGACTGCTGCTAACGGTTG | qRT-PCR |
| *CDKA2* | GCCTCATCTTGTCCATTTGT | CCCGCAATAAGGATCTTTCA | qRT-PCR |
| *CYCD4.2* | TGCCAGGAAGCGATTCAG | TTGCCTTTGTCGAGATGAGT | qRT-PCR |
| *CDKC1* | GTTGGTGGTGGCTATGGAGG | TGGATTCAGGGCAATACACG | qRT-PCR |
| *CYCU3.1* | GACGTTCGGAAGCTACTGC | GTGGTCCTAACCCTTGGTG | qRT-PCR |
| *RB2* | CTCGGCTATCTCGGTTTCC | CACTGTTTAGGCGGTTGTTT | qRT-PCR |
