# Supplementary material for: Identification of SMG3, a QTL Coordinately Controls Grain Size, Grain Number per Panicle, and Grain Weight in Rice
Source: Front Plant Sci. 2022 Apr 25;13:880919. doi: 10.3389/fpls.2022.880919 (PMC9085218; doi:10.3389/fpls.2022.880919)
Supplement: Supplementary file 2 [file Table_2.DOCX]

Supplementary Table 2 QTLs detected for grain size, grain weight and grain number per panicle in the F_2_ population

| Trait | QTL | Chr. | Interval | LOD | *A* | *R*^2^ (%) |
| --- | --- | --- | --- | --- | --- | --- |
| GL | *qGL3* | 3 | RM15087-RM15845 | 62.95 | 2.436 | 49.83 |
| GW | *qGW1* | 1 | RM1095-RM6840 | 3.61 | -0.085 | 15.08 |
| GW | *qGW3* | 3 | RM15087-RM15845 | 6.04 | -0.091 | 17.11 |
| GW | *qGW4* | 4 | RM5979-RM17470 | 3.06 | -0.068 | 9.04 |
| GW | *qGW5* | 5 | RM168-RM5140 | 7.38 | -0.086 | 14.64 |
| GW | *qGW7* | 7 | RM5436-RM5711 | 3.17 | -0.056 | 6.71 |
| GW | *qGW8* | 8 | RM3452-RM7631 | 3.53 | -0.073 | 13.11 |
| LWR | *qLWR3* | 3 | RM15087-RM15845 | 63.60 | 1.000 | 79.11 |
| LWR | *qLWR5* | 5 | RM168-RM5140 | 3.03 | 0.157 | 5.14 |
| LWR | *qLWR7* | 7 | RM5436-RM5711 | 3.01 | 0.121 | 3.00 |
| TGW | *qTGW3* | 3 | RM15087-RM15845 | 32.36 | 5.727 | 81.02 |
| GNP | *qGNP1* | 1 | RM1095-RM3281 | 4.80 | 23.496 | 20.91 |
| GNP | *qGNP2* | 3 | RM15087-RM3646 | 10.66 | -30.366 | 35.90 |
| GNP | *qGNP4* | 4 | RM16762-RM5979 | 2.77 | -16.067 | 9.10 |

GL, grain length (mm), GW, grain width (mm), LWR, the ratio of grain length to grain width, TGW, thousand grain weight (g). *A*, Additive effect of QTL, Positive value and negative value of additive effects represented the Zhong9 B (Z9B) and M494 alleles, respectively. *R*, variance explained by the QTL.
