# Supplementary material for: Identification of SMG3, a QTL Coordinately Controls Grain Size, Grain Number per Panicle, and Grain Weight in Rice
Source: Front Plant Sci. 2022 Apr 25;13:880919. doi: 10.3389/fpls.2022.880919 (PMC9085218; doi:10.3389/fpls.2022.880919)
Supplement: Supplementary file 3 [file Data_Sheet_1.docx]

**Supplementary Material**


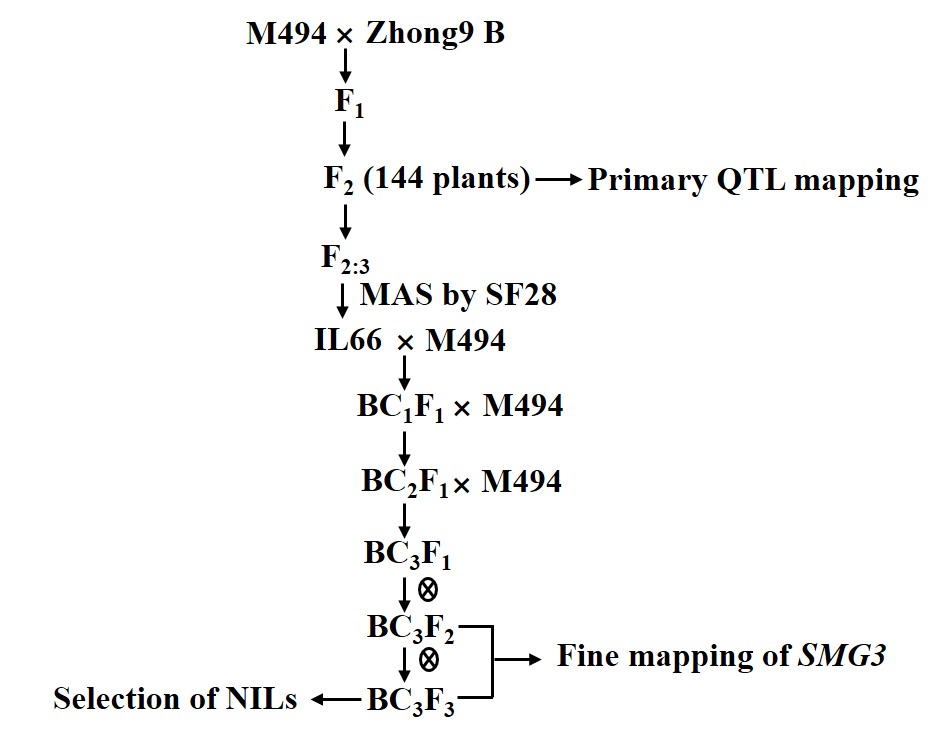


**Supplementary Figure 1. The flow chart of genetic mapping of SMG3 in this paper.**


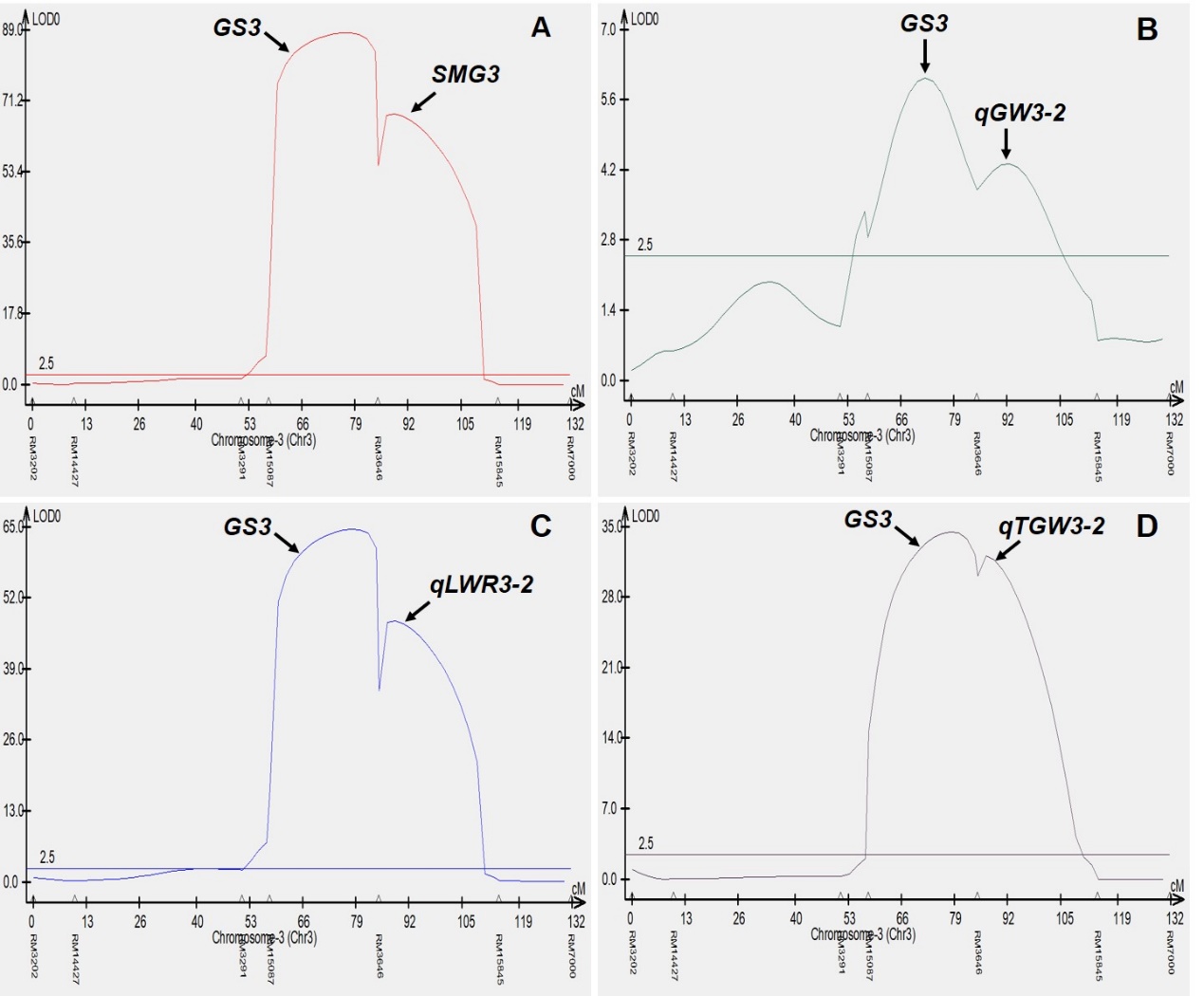


**Supplementary Figure 2. QTL scan for grain length (A), grain width (B), grain length to width ratio (C) and thousand grain weight (D) on chromosome 3 in the F_2_ population from the cross between Zhong9 B (Z9B) and M494.**

**
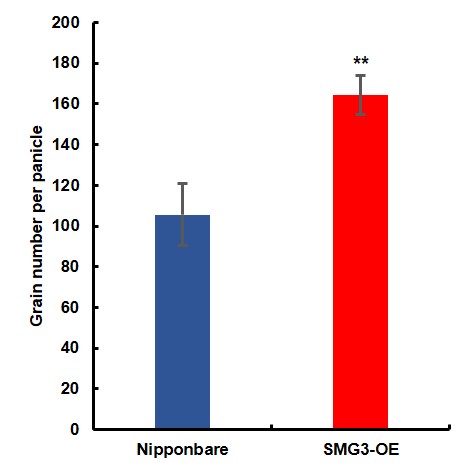
**

**Supplementary Figure 3. Comparison of the grain number per panicle between Nipponbare and the *SMG3* overexpression (OE) transgenic plants. Data are given as means ± SD. ** indicates *P* < 0.01 by Student’s *t*-test.**

**
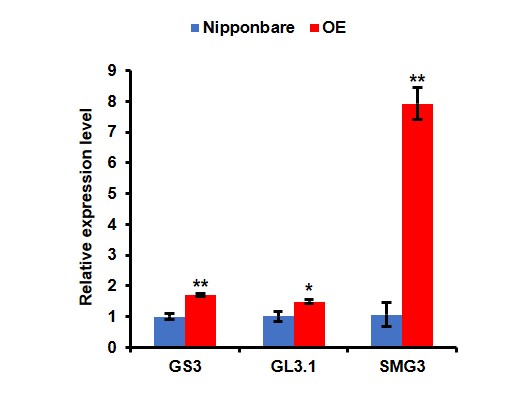
**

**Supplementary Figure 4. qPCR analysis of *GS3*, *GL3.1* and *SMG3* genes in the Nipponbare and *SMG3* overexpression (OE) transgenic plants. Data are given as means ± SD. * and ** indicate *P* < 0.05 and *P* < 0.01 by Student’s *t*-test.**

***
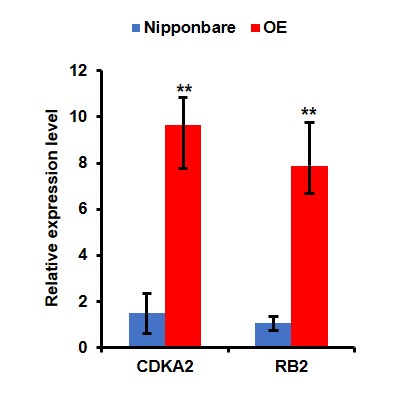
***

**Supplementary Figure 5. qPCR analysis of *CDKA2* and *RB2* genes in the Nipponbare and *SMG3* overexpression (OE) transgenic plants. Data are given as means ± SD. ** indicates *P* < 0.01 by Student’s *t*-test.**
